# A Case of Polymerase Chain Reaction (PCR)-Positive Urine Antigen and Culture-Negative Legionnaires’ Disease in an Immunosuppressed Patient

**DOI:** 10.7759/cureus.84984

**Published:** 2025-05-28

**Authors:** Matthew Rice, Ganesh Ram, Sagah Ahmed, Norman Bernstein

**Affiliations:** 1 Biomedical Sciences, Liberty University College of Osteopathic Medicine, Lynchburg, USA; 2 Internal Medicine, Mary Washington Healthcare, Fredericksburg, USA; 3 Infectious Disease, Mary Washington Healthcare, Fredericksburg, USA

**Keywords:** community-aquired pneumonia, immunocompromised, infectious disease medicine, legionella infection, urine antigen test

## Abstract

*Legionella *is an aerobic, intracellular, Gram-negative bacillus native to warm water sources and soils and is transmitted to humans via aerosols. While there are numerous different species of *Legionella*, the most commonly encountered is *Legionella pneumophila*, particularly serotype 1. This bacteriumnotably causes a mild, self-limited infection known as Pontiac fever, in addition to a more severe form of pneumonia calledLegionnaires' disease. Like any pneumonia, Legionnaires’ disease carries a mortality risk, making early and rapid detection crucial. This is of particular importance in immunosuppressed patients, as they possess a weakened cell-mediated defense. As with any intracellular organism, host immune defense plays a crucial role in the protection against the *Legionella* species, as cell-mediated immunity is vital in restricting intracellular growth. For detection, the least invasive and most commonly used test for *Legionella *is a urine antigen test (UAT). However, one major limitation of the UAT is that it only detects *Legionella pneumophila* serotype 1 antigen, which can lead to false negatives. Cultures, typically obtained from respiratory secretions, are usually the next diagnostic test used due to their ability to detect more species of *Legionella* than just *Legionella* ​​​​​​*pneumophila* serotype 1. However, while broader than UAT, cultures lack sensitivity and can take days to show any growth. Given the limitations of these first-line diagnostic tests, the following case report highlights that further testing with polymerase chain reaction (PCR) may be necessary to definitively rule out Legionnaires’ disease in already high-risk, immunosuppressed patients when clinical suspicion is strong.

## Introduction

Legionnaires' disease is a sometimes fatal pneumonia that results from *Legionella *infection. The presentation of Legionnaires' disease varies, often including typical pneumonia features such as fever, cough, and radiographic evidence of pneumonia, in addition to more unique features such as diarrhea, encephalopathy, and lab abnormalities like hyponatremia [1]. High clinical suspicion should be given to patients presenting with these atypical features in addition to the findings commonly seen in community-acquired pneumonia. Thorough history-taking is also vital, as identifying all possible exposures to the bacteria is crucial. With prompt identification, Legionnaires' disease is a treatable illness that typically resolves with the use of macrolides or respiratory fluoroquinolones, which are first-line treatments [1]. 

Due to immunosuppressed patients being at particularly high risk for *Legionella *infections, the CDC recommends that all immunosuppressed patients with features of pneumonia be tested for *Legionella *[2].

In its most recent surveillance report [3], the CDC reported only 16,452 confirmed cases of Legionnaires’ disease in the United States between 2018 and 2019. Of the 16,452 confirmed cases, 15,737, or 97%, were detected by a positive urine antigen test (UAT). Of the remaining cases, 708 were confirmed using culture, and the remaining seven cases by serology. Among the 708 culture-confirmed cases, respiratory secretions were the most common specimen collected at nearly 76% [3].

The following is a case presentation of an immunosuppressed patient who presented with a clinical picture consistent with pneumonia, in addition to many of the unique symptoms that are seen in *Legionella *infection, as previously discussed. The patient was evaluated for possible Legionellosis and found to have a negative UAT and negative respiratory culture but a positive respiratory polymerase chain reaction (PCR) for *Legionella*. This case presentation highlights the importance of utilizing all of the initially recommended diagnostic tools for *Legionella*, in addition to respiratory PCR in immunosuppressed patients for whom there is a high clinical suspicion of Legionnaires’ disease.

## Case presentation

A 53-year-old female patient with a past medical history of systemic lupus erythematosus (SLE), on mycophenolate mofetil and chronic prednisone, presented to the emergency department (ED) with a four-day history of fever, nausea, vomiting, diarrhea, shortness of breath, fatigue, and cough. The week prior, she reported significant bouts of diarrhea that caused her to present to another ED. During her initial visit to that emergency department, the patient received fluids and electrolyte replenishment but was later discharged after testing negative for common viral and bacterial causes. Following discharge, the patient’s symptoms progressively worsened, with persistent fever and daily episodes of diarrhea and vomiting. Four days after initial discharge, the patient presented to our emergency room after experiencing a seizure at home. On arrival to our ED, the patient’s vitals were notable for a temperature of 103.1℉ and an oxygen saturation of 87% on room air. Initial laboratory studies revealed several abnormalities, as summarized in Table 1.

**Table 1:** The patient's laboratory results on presentation

Laboratory test	Patient value	Normal range	Units
White blood cell count (WBC)	23.83	4.0 – 10.0	x10³/µL
Magnesium	2.6	1.7 – 2.2	mg/dL
Sodium	133	135 – 145	mmol/L
Creatinine	1.7	0.6 – 1.2	mg/dL
Potassium	2.1	3.5 – 5.0	mmol/L

**Figure 1:**
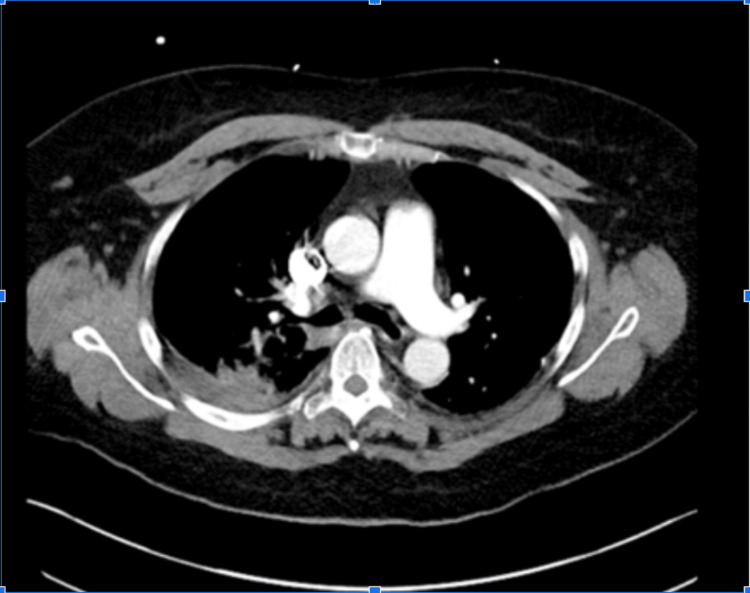
Chest CT performed on Day 1

Imaging on presentation using computed tomography (CT) chest without contrast was notable for dense opacities of the right lower lobe and right hilar region consistent with right lower lobe pneumonitis (Figure 1). Blood cultures were obtained before she was started on empiric broad coverage treatment in the emergency department with intravenous (IV) vancomycin, IV cefepime, and IV azithromycin, given her immunosuppressed status. The patient was then admitted for further evaluation and care. Following admission to the internal medicine service, she was worked up for numerous infectious etiologies. Microbiological tests, in addition to blood cultures, included sputum culture, viral respiratory panel, and urine antigens for *Streptococcus pneumoniae *and *Legionella pneumophila*, all of which were negative.

**Figure 2:**
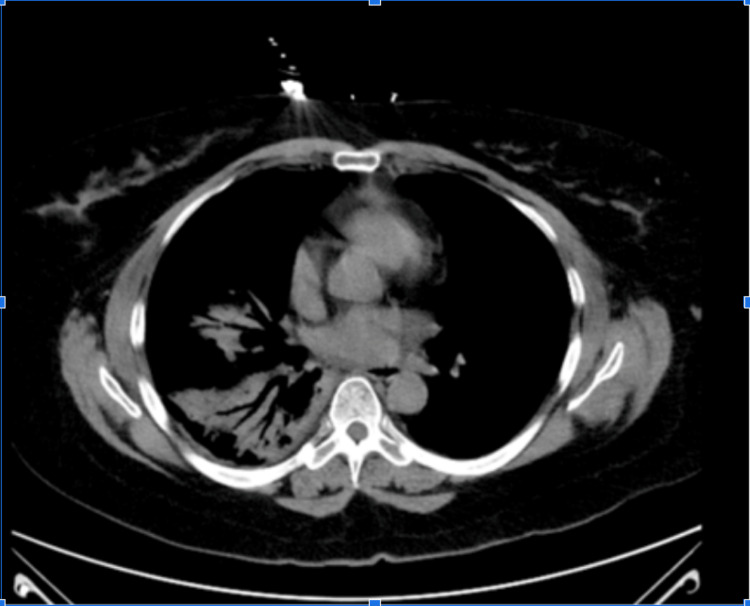
CT angiography performed on on Day 5

Despite continued antibiotic treatment, the patient continued to have worsening shortness of breath. CT angiography (CTA) was done on hospital day five and showed progression of the right lower lobe pneumonia, which led to the infectious diseases (ID) team being consulted (Figure 2). 

Given the presentation of pneumonia in an immunosuppressed patient with diarrhea and hyponatremia, the ID team recommended a pulmonology consult to perform a bronchoscopy with bronchoalveolar lavage (BAL). While awaiting the bronchoscopy, the patient began to improve clinically, but given the immunosuppressed state of the patient and the slow rate of recovery, the decision was made to proceed with the bronchoscopy. The BAL specimen was submitted for Gram stain, as were cultures for aerobic organisms, anaerobic organisms, acid-fast bacteria (AFB), fungal organisms, and *Legionella*. All of these cultures were negative for any growth. The BAL specimen was then analyzed using PCR, which was positive for *Legionella pneumophila*. As a result, the patient was treated with azithromycin 500 mg for 14 days at the recommendations of the ID team and ultimately made a complete recovery.

## Discussion

Overall, Legionnaires' disease generally proves to be straightforward to treat. However, detection of *Legionella *species apart from *Legionella pneumophila* serotype 1 can be challenging at times, given its rarity. Of the reported cases of Legionnaires' disease, it is estimated that 80% to 90% are caused by *Legionella pneumophila* serotype 1 [1]. Current recommendations from the CDC for diagnosing Legionnaires' disease include using both UAT and specimen culture, as culturing allows for broader detection beyond *Legionella pneumophila* serotype 1 [4]. While culture and UAT prove to be very specific tests, at 100% and 95% to 100% [4], respectively, there are known limitations. For UAT, the main limitation remains its inability to detect species beyond *Legionella pneumophila* serotype 1. Cultures, while specific, have test sensitivity ranges from 20% to 80% [4], likely due to the fastidious nature of *Legionella*. A known limitation of culturing *Legionella *is that cultures are often not performed until after patients have started antibiotics, which can interfere with organism isolation. In addition, it takes three to five days for *Legionella *to form visible colonies [5], which may not be feasible when dealing with a possibly life-threatening illness. Respiratory PCR offers the greatest combined sensitivity and specificity at 95% to 99% and >99%, respectively [4]. Acceptable specimens include sputum, BAL fluid, lung tissue, bronchial washings, and pleural fluid. Overall, respiratory PCR proves to be a successful tool in the detection of *Legionella*, as it is capable of detecting all species [6]. In the case of our patient, the empiric atypical pneumonia coverage she initially received with azithromycin was vital in her recovery. Overall, this case highlights the importance of initiating empiric therapy that covers atypical organisms such as *Legionella*, *Chlamydia pneumoniae*, and *Mycoplasma pneumoniae *in immunosuppressed patients presenting with features of pneumonia. In addition, the timely ordering of appropriate diagnostic tests, including respiratory PCR, is crucial to guide targeted treatment.

## Conclusions

In the setting of an immunosuppressed patient with features concerning for pneumonia, prompt diagnostic testing is crucial for early detection and reduction in mortality. Specifically, particular consideration should be given to *Legionella *in immunosuppressed patients with signs and symptoms concerning for Legionnaires' disease. While testing with UAT and culture for *Legionella *is recommended and sufficient in most cases, further testing using respiratory PCR should always be considered if clinical suspicion remains high in spite of initially negative tests and lack of clinical improvement.
